# Police suspect interviews with autistic adults: The impact of truth telling versus deception on testimony

**DOI:** 10.3389/fpsyg.2023.1117415

**Published:** 2023-03-22

**Authors:** Ralph Bagnall, Aimee Cadman, Ailsa Russell, Mark Brosnan, Marco Otte, Katie L. Maras

**Affiliations:** ^1^Centre for Applied Autism Research, Department of Psychology, University of Bath, Bath, United Kingdom; ^2^Vrije Universiteit Amsterdam, Amsterdam, Netherlands

**Keywords:** investigative interviewing, autism (ASD), deception, social perception, virtual environment

## Abstract

Investigative interviews by police are socially and cognitively demanding encounters, likely presenting significant challenges to those on the autism spectrum. Behavioral and communication differences mean that autistic people may also be more likely to be perceived as deceptive in the context of an investigative interview. In the present study, 32 autistic and 33 (age and IQ-matched) non-autistic adults took part in a novel virtual burglary scenario in either an ‘innocent’ or ‘guilty’ condition. In a subsequent mock-police interview, innocent suspects were instructed to tell the truth about what they did, while guilty suspects were instructed to lie in order to convince the interviewer of their innocence. In the mock-interviews, innocent autistic mock-suspects reported fewer details that would support their innocence than non-autistic mock-suspects, although both innocent and guilty autistic and non-autistic mock-suspects reported similar levels of investigation-relevant information and had similar levels of statement-evidence consistency. In post-interview questionnaires, innocent and guilty autistic mock-suspects self-reported greater difficulty in understanding interview questions, higher anxiety and perceived the interview as less supportive than non-autistic participants. Implications for investigative interviewing with autistic suspects and cues to deception are discussed.

## Introduction

Whether a suspect appears to be telling the truth or lying during an investigative interview has far-reaching implications; from the perceived reliability of their statement by police to how they are viewed by jurors in court (Denault, 2020; Haworth, 2020). However, accuracy for detecting deception is broadly at chance level, often based upon faint or unreliable nonverbal and paraverbal cues such as eye contact and vocal pitch (Bond and DePaulo, 2006; Sporer and Schwandt, 2006). Verbal deception cues have proved a more reliable and promising direction for investigative interviewing research. Richness of detail is one such verbal indicator of veracity (Nahari, 2016) as liars use self-regulation strategies such as keeping a story simple and avoiding verifiable details that could reveal deceit (Hartwig et al., 2014; Nahari, 2018). Further, in the criminal justice system (CJS), evidence typically links a suspect to an alleged offence, meaning a suspect’s account can be compared against available evidence (Police and Criminal Evidence Act: PACE. Home Office, 1984/2008). Thus, lying suspects may display verbal deception cues such as contradicting this evidence (‘statement-evidence inconsistencies’) (Hartwig et al., 2005; Vredeveldt et al., 2014), viewed as indicative of deception by investigative officers in the field (Deeb et al., 2018).

However, the impact of such verbal deception cues within investigative interviews is, to date, based upon neurotypical population samples. Thus, whether such deception cues are applicable to neurodivergent adults is yet to be examined. Concerningly, individuals on the autism spectrum (henceforth autism) appear to be overrepresented in the CJS (Justice Inspectorates, 2021). Autism is a lifelong neurodevelopmental condition characterized by persistent difficulties with social communication and interaction, as well as restricted and repetitive behaviors, interests and activities (American Psychiatric Association, 2013; World Health Organization, 2018). Relatedly, social communication and memory differences in autism present substantial challenges for providing best evidence’ during investigative interviews (Maras, 2021). Autistic mock-witnesses often provide less detailed free-recall accounts (Maras and Bowler, 2011, 2012; Henry et al., 2020) due in part to autism-common difficulties with episodic memory retrieval, exacerbated by insufficiently specific, structured questioning (Maras, 2021). More broadly, autistic individuals may produce less coherent and causally connected narrative versions of events with fewer key contextualizing details (Barnes and Baron-Cohen, 2012; Baixauli et al., 2016; but see Henry et al., 2020). Indeed, autistic adults more often fail to recognize and report extricating details that would help demonstrate their innocence of mock-criminal offences (Young and Brewer, 2020). Thus, even when being truthful, autistic suspects may display verbal cues associated with deception in neurotypical populations, such as statement-evidence inconsistencies (Vredeveldt et al., 2014), a lack of verifiable extricating information (Nahari, 2018) and sparsely detailed accounts (Vrij et al., 2014). Verbal responses containing insufficient information may be interpreted as evasive or deceptive, leading to repeated questioning and challenges by investigators (Gudjonsson et al., 2007) which, in turn may lead to further breakdown in communication (O’Mahony et al., 2012) and even false confessions (Gudjonsson, 2021). These issues are likely to be further exacerbated by the stress of a suspect interview experience, as autistic people may experience investigative interviews as highly socially and cognitively demanding (Herrington and Roberts, 2012; Maras et al., 2020).

Understanding the verbal behavior of autistic suspects who are actively deceptive is also crucial for effective investigative interviewing practice. While there is a substantial body of research showing that autistic children have difficulty with lying, relatively little is known about deception in autistic adulthood (Bagnall et al., 2022). Over the past few years, there have, however, been high-profile criminal cases in the United Kingdom (UK) in which autistic defendants have deceived others (e.g., Murray, 2020; De Simone, 2021). Like non-autistic individuals, some autistic adults without co-occurring intellectual disability tell verbal lies for self-protective purposes (Davidson and Henderson, 2010; Jaarsma et al., 2012) and can successfully deceive in computerized paradigms (van Tiel et al., 2021). Autistic adults also report an inclination to lie in everyday situations comparable with non-autistic adults, though such deception may require greater cognitive effort than neurotypical peers (Bagnall et al., under review). Deception, during even mock-suspect interviews, can be highly cognitively demanding (Caso et al., 2005). Common (though not universal) autism difficulties in taking others’ perspectives—or Theory of Mind (ToM: Baron-Cohen, 1997; but see Milton, 2012)—and social decision-making (Woodcock et al., 2020; Brosnan and Ashwin, 2022) may suggest that autistic adults’ verbal deception cues are more pronounced than those of non-autistic adults. Indeed, while many autistic children can and do tell spontaneous verbal lies, they tend to have greater difficulty than non-autistic children maintaining these lies during subsequent follow-up statements (Li et al., 2011). Identifying how verbal deception cues are displayed by (both truthful and lying) autistic mock-suspects is crucial for the development of best practice investigative interviewing.

In summary, socio-cognitive and sensory processing differences in autism raise numerous concerns relating to the investigative interviewing of autistic suspects. Ensuring that police suspect interviews are conducted fairly and ethically requires understanding if (and how) autistic peoples’ accounts are affected, and if this depends on whether they are being truthful or deceptive. Recognizing how those on the autism spectrum experience investigative interviews is also crucial for identifying relevant areas of support (e.g., supportive interviewing practices or adjustments to custody – see Holloway et al., 2020; Maras et al., 2020). We address these issues in the present study. We predicted that, during a mock-suspect interview, autistic adults’ deceptive accounts would present more pronounced verbal cues to deception (i.e., greater inconsistencies, sparser accounts) than those of non-autistic adults. We also expected that autistic adults’ truthful accounts would more frequently display verbal deception cues (i.e., greater inconsistencies, sparser accounts and fewer verifiable extricating details) than neurotypical adults’ truthful accounts. We also anticipated that experiencing the mock-suspect interview process would be more challenging for autistic than non-autistic adults (i.e., difficulty understanding questions, level of anxiety, how supported they feel).

## Methods

### Participants

A power analysis using G*Power (Faul et al., 2007) indicated that a total sample size of 64 would provide 80% power to detect a medium-large effects of interview performance. This is consistent with previous studies reporting medium (partial *η*^2^ = 0.07) to large (*d* = 0.94) effect sizes in the difference of verbal information provided by autistic and non-autistic participants during mock-forensic interviews (Maras et al., 2020; Young and Brewer, 2020).

Participants were recruited *via* research participant databases, as well as physical and digital advertisements. All participants stated that they met the eligibility criteria of normal or corrected to normal vision and hearing, adequate computer ability, fluency in spoken and written English and no previous real-life experience of a police suspect interview. The final sample was comprised 32 autistic participants (*M =* 35.25 years, SD = 14.93), including 13 females, 16 males and three non-binary individuals and 33 non-autistic participants (*M =* 35.15 years, SD = 17.55) including 21 females and 12 males. Autistic participants provided documentary evidence of their formal autism diagnosis meeting Diagnostic and Statistical Manual of Mental Disorders (4th ed.; DSM-IV; American Psychiatric Association, 2000) or DSM-5 criteria (American Psychiatric Association, 2013). As expected, the autistic group (*M =* 33.97, SD = 6.54) scored significantly higher than non-autistic participants (*M =* 15.70, SD = 8.76) on the Autism Spectrum Quotient (AQ-50; Baron-Cohen et al., 2001), *t*(63) = 9.51, *p* < 0.001, *d* = 2.36. The autistic group were significantly above the proposed autism threshold score of 26 (Woodbury-Smith et al., 2005), *t*(31) = 6.90, *p* < 0.001, Hedges’ *g =* 1.22, and the non-autistic group were significantly below this threshold, *t*(32) = −6.76, *p* < 0.001, Hedges’ *g = −*1.78. Four participants who considered themselves to be non-autistic scored above 26 on the AQ-50 (scores of 26, 28, 34, and 40). Three autistic participants had AQ-50 scores below the 26 thresholds (scores of 18, 23, and 25). In the interest of reflecting diversity in autistic and neurotypical samples, these participants were retained in the dataset and analysis.^1^

As both age (Debey et al., 2015) and cognitive ability (Sarzyńska et al., 2017; Littrell et al., 2021; though see Wright et al., 2012) have each been associated with deceptive behavior, we assessed whether the groups were matched on these characteristics. We also examined participants’ previous level of experience playing computer games,^2^ as the mock-criminal and non-criminal tasks were performed within an interactive virtual environment (see ‘Procedure’ section of Method). A series of two-way analyses of variance (ANOVAs) were performed to examine group characteristics of age, IQ and previous gaming experience (see Table 1). There were no main effects of Group (autistic vs. non-autistic) or Condition (innocent vs. guilty), or a Group X Condition interaction for age (all *ps* > 0.921, all partial *η*^2^s < 0.001). While there was no main effect of Group or Group X Condition interaction for IQ (*ps > 0*.170, partial *η*^2^s < 0.031), there was a significant main effect of Condition in which participants in the Guilty condition (*M =* 120.96) had significantly higher IQ scores than participants in the Innocent condition (*M* = 114.96), *F*(1, 61) = 5.36, *p* = 0.024, partial η^2^ = 0.081. There were no main effects of Group or Condition, or a Group X Condition interaction for gaming experience (all *ps* > 0.068, all partial *η*^2^s < 0.053). However, controlling for IQ led to a significant main effect of gaming experience on Group and Condition (*p*s < 0.047, partial *η*^2^s > 0.064), but no Group X Condition interaction (*p* = 0.610, partial *η*^2^ = 0.004). Consequently, we controlled for gaming experience as well as IQ when comparing innocent and guilty conditions in the subsequent analyses.

**Table 1:** Autistic and non-autistic group mean scores for age, IQ, and gaming experience within interview conditions (standard deviations in parenthesis).

	Autistic adults (*n =* 32)		Non-autistic adults (*n =* 33)	
Innocent (*n* = 32)	(*n* = 17)		(*n* = 15)	
Age	34.88	−15.11	35.13	−16.41
IQ^a^	113.47	−11.64	116.46	−11.23
Gaming experience	2.65	−1.54	2.2	−1.27
Guilty (*n* = 33)	(*n* = 15)		(*n* = 18)	
Age	35.67	−15.24	35.17	−18.92
IQ^a^	118.86	−8.77	123.05	−9.64
Gaming experience	2.33	−1.4	1.61	−0.79

^a^IQ was measured using vocabulary and matrix reasoning subtests on the Wechsler Abbreviated Scale of Intelligence (WASI-II: Wechsler, 2011).

The study received ethical approval from the Psychology Ethics committee at the University of Bath (21–239).

### Procedure

#### Virtual environment

The study used an experimental paradigm in which participants either undertook a simulated ‘criminal’ or ‘non-criminal’ task in a virtual environment (VE). To our knowledge, this is the first investigative interviewing study to use VE technology. We adapted a VE originally developed by Nee et al. (2019), especially for the purposes of the present study. The original VE was developed and for this project updated using Unity Pro (2019) as the main development platform. The geometry of different sections within the VE was either created especially for the present study or purchased from the Unity Asset Store and adapted where required. For the creation and animation of humanoid avatar, we used the Character Creator (v3) and iClone (v7) software by Reallusion. The flow of the application through the different sections, on-screen messaging and data logging were accomplished through custom-designed C# code inside Unity Pro.

**Figure 1:**
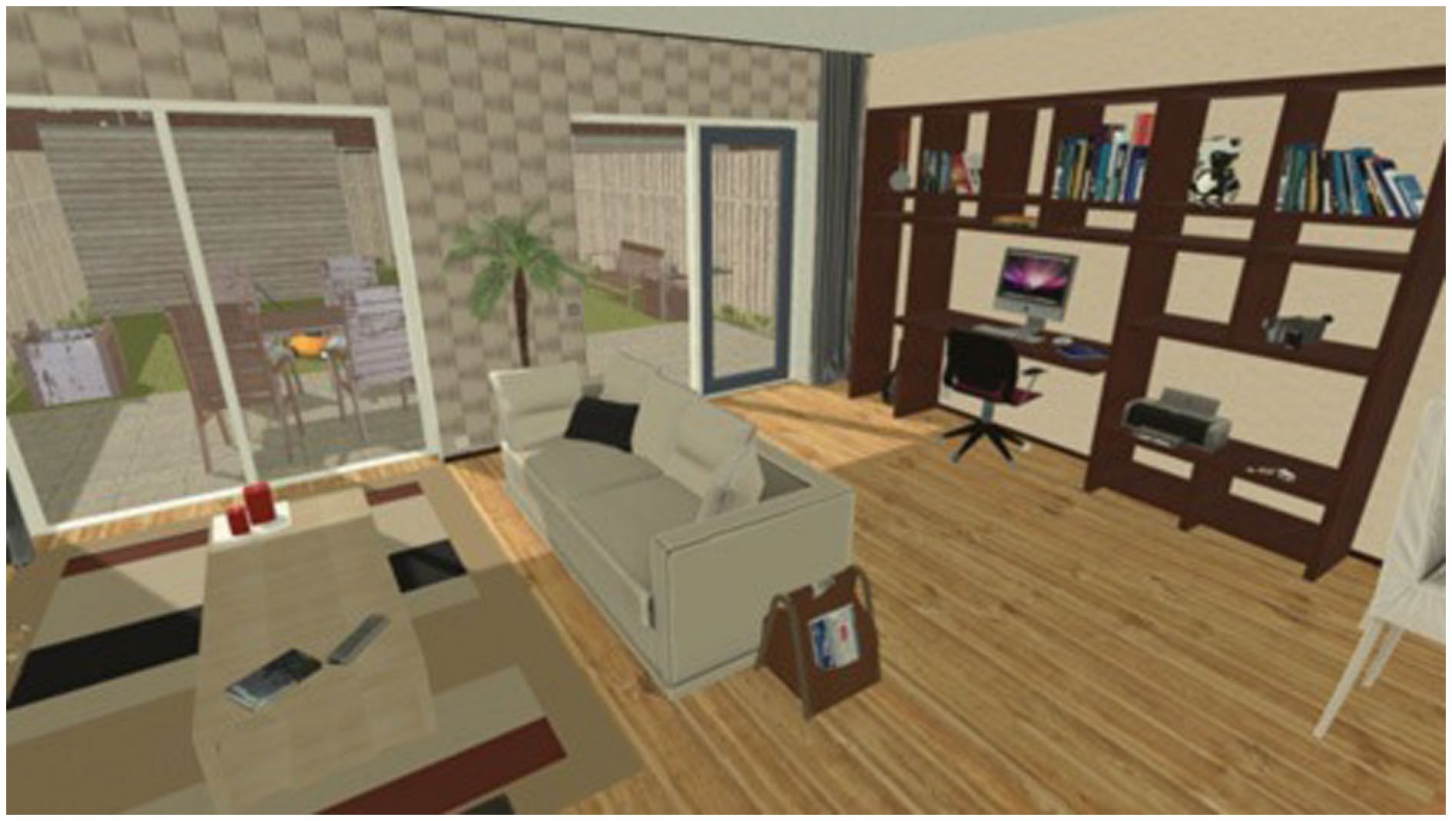
Image from within the virtual environment.

The adapted VE was piloted throughout the development to ensure usability. In the final VE, participants explored three distinct environments: a city, a suburban area and a residential property. The VE was presented using a high-performance gaming laptop computer, with headphones for immersive environmental audio (e.g., footsteps, passing cars, birdsong, etc.). Figure 1 presents an image from within the VE.

#### VE task and post-task

Participants took part in the study individually in a dedicated laboratory space at the University of Bath. Participants were randomly allocated to either a *guilty/criminal* or *innocent/non-criminal* VE condition with associated task and instructions^3^ (appearing in-game as ‘text messages’). In the both the *guilty* and the *innocent* conditions, participants received seven key text messages within the VE. In the *guilty* condition the messages were from their purported ‘criminal collaborator’ whose instructions participants follow to steal a laptop from a residential property. In the *innocent* condition the messages were from a ‘friend’ who asks the participant to locate (though not touch or remove) a missing laptop. Following these instructions created seven pieces of incriminating evidence against the participants, identical in both *innocent* and *guilty* conditions (see Appendix 1).

Post-VE task, participants completed Lessiter et al.’s (2001) ITC-Sense of Presence Inventory (ITC-SOPI) to measure level of immersion during the VE, the AQ-50 and rated their frequency of gaming experience (as reported earlier) in Qualtrics. The first author then administered the WASI-II vocabulary and matrix reasoning subtests.

#### Mock-suspect interview

Participants (in innocent and guilty conditions) received written instructions stating they were to be interviewed as a suspect in a burglary investigation and that they needed to attempt to convince the interviewer of their innocence (e.g., Hartwig et al., 2014). Participants were informed that it was likely that the police held some evidence against them, so they should establish a plausible story. To encourage motivation, participants were instructed that if they were successfully able to convince the interviewer, they would be entered into a lottery draw to win £50 in Amazon vouchers (in actual fact, all participants were entered into the draw). Participants had 10 min to prepare before being escorted to a separate interview room.

All interviews were conducted by the second author (then blind to all research questions, hypotheses and participants’ diagnosis and veracity condition), who received training by the first author in investigative interviewing practice. The interviews followed a novel script which was responsive to the content of participants’ verbal accounts and was based upon UK investigative interviewing protocol.^4^ All the interviews proceeded in three phases: (1) *obtain mock-suspects’ initial account*; (2) *probe questions of topics from initial account* and (3) *disclosure of incriminating evidence* (see Appendix 2) while being audio and video recorded.

#### Post-interview task

Participants completed a Qualtrics questionnaire on a 7-point scale (1 = Not at all; 4 = Neutral; 7 = Completely) which provided the dependent variables (DVs) to assess *interview motivation*: (1) participants’ motivation to appear convincing; (2) the truthfulness of participants’ accounts and (3) the deceptiveness of participants’ accounts and *interview experience* (1) the difficulty of the interviewer’s questions; (2) participants’ level of anxiety during the interview and (3) how supported participants’ felt to provide a full account. Participants were also asked to rate the extent to which they remembered the details of the VE task on the aforementioned 7-point scale. At the end of the study, all participants were fully debriefed and were reimbursed at £10 per hour (the study typically lasted 90 min).

#### Interview coding

Interviews transcripts were coded to produce three DVs for *interview performance.*

(1) *Statement-evidence consistency* (total scores = 0–7) measured how consistent participants’ (in innocent and guilty conditions) accounts were with the seven pieces of incriminating evidence (see Hartwig et al., 2005). For example, if a participant described getting off the bus near the burgled property, they would score 1 point as this was consistent with the evidence held by the interviewer (i.e. CCTV footage from bus stop). If a participant failed to mention or denied getting off the bus, they would score 0 for that piece of incriminating evidence.
*Extricating information* (total scores = 0–7) measured whether participants (innocent condition only) explained that each of the seven pieces of incriminating evidence were due to their ‘friend’ having asked them to perform those actions. For example, a participant would score 1 point if they specified their friend had asked them to enter the property. If the participant failed to mention this, they would score 0 for this piece of evidence linking them to the crime scene.
*(2) Investigation-relevant information (IRI)* (total score range: 20–223) measured the level of detail in participants’ innocent and guilty mock-suspect accounts. The ‘PALIT’ (Person, Action, Location, Item, Temporal) coding scheme was used (see Oxburgh et al., 2012; Farrugia and Gabbert, 2020a). For example, ‘*I went to the bus stop at 1.30 pm’* (1 × Action; 1 × Location; 1 × Temporal) ‘*and saw a woman wearing a black coat’* (1 × Action; 1 × Person; 2 × Item). Each item of information was only coded once with all repetitions ignored. PALIT details were summed to produce a total IRI score for each participant.

Twenty per cent of the interviews (*n =* 14) were double-coded with intraclass correlations performed for statement-evidence consistency (*r = 0*.929, *p < 0*.001; *α* = 0.929), extricating information (*r = 0*.945, *p = 0*.008; *α* = 0.945) and quantity of investigation-relevant information (IRI) (*r = 0*.958, *p < 0*.001; *α* = 0.978), all of which showed excellent interrater reliability (Cicchetti, 1994).

### Statistical analysis

All statistical analyses were performed using SPSS (version 28). All analyses comparing innocent and guilty conditions controlled for participants’ IQ and level of previous gaming experience as covariates. First, *task immersion, duration and motivation* (see Table 2) was assessed using a series of two-way ANCOVAs in a 2 (Group: autistic vs. non-autistic) X 2 (Condition: innocent vs. guilty) design. Second, we examined *interview performance* (i.e., statement-evidence consistency; extricating information; IRI detail). Statement-evidence consistency and IRI detail were each investigated using a two-way ANCOVA. A *t-*test was conducted to examine whether quantity of extricating information differed between autistic and non-autistic groups (innocent condition only). Finally, *interview experience* (i.e. difficulty of questioning; interview anxiety and perception of support) was analyzed using a series of two-way ANCOVAs.

**Table 2:** Autistic and non-autistic group estimated mean scores in task immersion, duration and motivation.

	Autistic adults (*n =* 32)				Non-autistic adults (*n =* 33)			
	M_adj_	SE	BCa 95% CI		M_adj_	SE	BCa 95% CI	
			Lower	Upper			Lower	Upper
Innocent (*n =* 32)								
ITC engagement	3.33	0.21	2.92	3.81	3.53	0.11	3.27	3.76
ITC ecological validity	2.75	0.22	2.33	3.15	3.19	0.22	2.78	3.59
ITC negative effects	2.57	0.24	2.13	3.09	2.01	0.26	1.53	2.5
ITC spatial presence	2.88	0.23	2.46	3.35	3.1	0.14	2.83	3.38
VE task memory	5.74	0.18	5.4	6.1	5.87	0.27	5.19	6.34
Interview duration ^b^	971.64	100.9	790.38	1189.59	894.92	59.2	778.95	1017.24
Interview motivation	5.63	0.32	4.87	6.31	5.73	0.33	4.99	6.33
Interview truthfulness	6.76	0.15	6.5	7.05	6.95	0.09	6.76	7.15
Interview deceptiveness	1.31	0.18	1	1.68	1.14	0.12	0.96	1.37
Guilty (*n =* 33)								
ITC engagement	3.26	0.15	2.95	3.56	3.32	0.17	3.03	3.64
ITC ecological validity	3.01	0.23	2.59	3.43	2.86	0.21	2.44	3.3
ITC negative effects	2.29	0.23	1.8	2.83	2.27	0.28	1.77	2.81
ITC spatial presence	2.82	0.19	2.4	3.2	2.93	0.21	2.52	3.36
VE task memory	5.59	0.29	4.95	6.09	5.7	0.2	5.29	6.09
Interview duration	746.07	82.56	616.57	908.51	646.41	62.85	519.66	770.01
Interview motivation	5.49	0.32	4.87	6.13	5.95	0.28	5.35	6.43
Interview truthfulness	3.11	0.36	2.42	3.82	3.13	0.35	2.47	3.79
Interview deceptiveness	5.28	0.3	4.59	5.92	5.24	0.23	4.74	5.72

BCa bootstrapped standard errors (SEs) and confidence intervals (CIs) for M_adj_ scores based on 1,000 resamples. ^a^Gaming experience controlled for with IQ only. ^b^Interview length is presented in seconds. ITC-Sense of Presence Inventory; VE, Virtual environment.

Data inspection revealed one significant outlier in truthfulness scores, two significant outliers in VE task memory and one significant outlier in extricating information scores (>3 standard deviations from mean). The overall pattern of results remained whether these outliers were included or excluded from the analysis, so they were therefore retained in the dataset. Non-normal distribution was observed in a number of variables, which a series of transformations failed to substantially improve. Heterogeneity of variance was also detected in statement-evidence consistency scores (Levine’s test, *p* < 0.001). Accordingly, bootstrapped 95% Bias corrected accelerated (BCa) confidence intervals (CIs) for estimated group means and/or mean differences were produced to account for violations (Field and Wilcox, 2017).

## Results

### Immersion, duration, and motivation

We first examined task immersion, duration and motivation (see Table 2). Innocent participants’ interviews were longer than guilty participants’ interviews, *F*(1, 59) = 8.95, *p* = 0.004, partial *η*^2^ = 0.132, *M* difference = 237.05 s, BCa 95% CI (78.49, 395.60). Innocent participants self-reported as being more truthful than guilty participants, *F*(1, 59) = 169.85, *p* < 0.001, partial *η*^2^ = 0.742, *M* difference = 3.73, BCa 95% CI (3.16, 4.31). Guilty participants self-reported as being more deceptive in the interview than innocent participants, *F*(1, 59) = 299.12, *p* < 0.001, partial *η*^2^ = 0.835, *M* difference = 4.03, BCa 95% CI (3.57, 4.50). All other main effects of diagnostic group and veracity condition and Group X Condition interactions were non-significant (*ps* > 0.175, partial *η*^2^s < 0.083). Therefore, autistic and non-autistic participants (across veracity conditions) were well matched in criminal and non-criminal VE task immersion, memory of the VE task and motivation to appear convincing during the interview. This indicates that our experimental manipulations (e.g., guilty participants needing to lie during the mock-suspect interview) were effective.

### Interview performance

Next, we compared autistic and non-autistic participants’ interview performance in guilty and innocent conditions (see Table 3).

**Table 3:** Autistic and non-autistic group estimated mean scores for interview performance and experience.

	Autistic adults (*n =* 32)				Non-autistic adults (*n =* 33)			
	M_adj_	SE	BCa 95% CI		M_adj_	SE	BCa 95% CI	
			Lower	Upper			Lower	Upper
Innocent (*n =* 32)								
Evidence consistency	6.31	0.24	5.78	6.79	6.84	0.13	6.52	7.07
Extricating information^a^	5.71	0.37	4.95	6.42	6.73	0.15	6.41	7.00
IRI detail	118.23	11.67	97.01	143.33	147.24	9.29	128.99	165.06
Difficulty of questioning	3.99	0.41	3.11	4.69	3.07	0.47	2.09	3.95
Interview anxiety	4.57	0.35	3.86	5.23	3.36	0.47	2.43	4.26
Perception of support	4.09	0.38	3.31	4.91	5.25	0.30	4.62	5.88
Guilty (*n =* 33)								
Evidence consistency	4.08	0.49	2.95	5.07	4.50	0.49	3.42	5.41
IRI detail	80.82	11.00	60.93	102.07	74.62	9.17	57.70	90.99
Difficulty of questioning	4.51	0.46	3.51	5.38	3.25	0.29	2.67	3.79
Interview anxiety	5.00	0.42	4.03	5.77	4.54	0.32	3.88	5.22
Perception of support	4.79	0.44	3.88	5.68	5.34	0.31	4.71	5.92

BCa bootstrapped standard errors (SEs) and confidence intervals (CIs) for M_adj_ scores based on 1,000 resamples. ^a^Extricating information was only measured in the innocent condition (therefore not controlled for IQ or gaming experience), so original means and SE are presented. IRI: Investigation-relevant information.

#### Statement-evidence consistency

**Figure 2:**
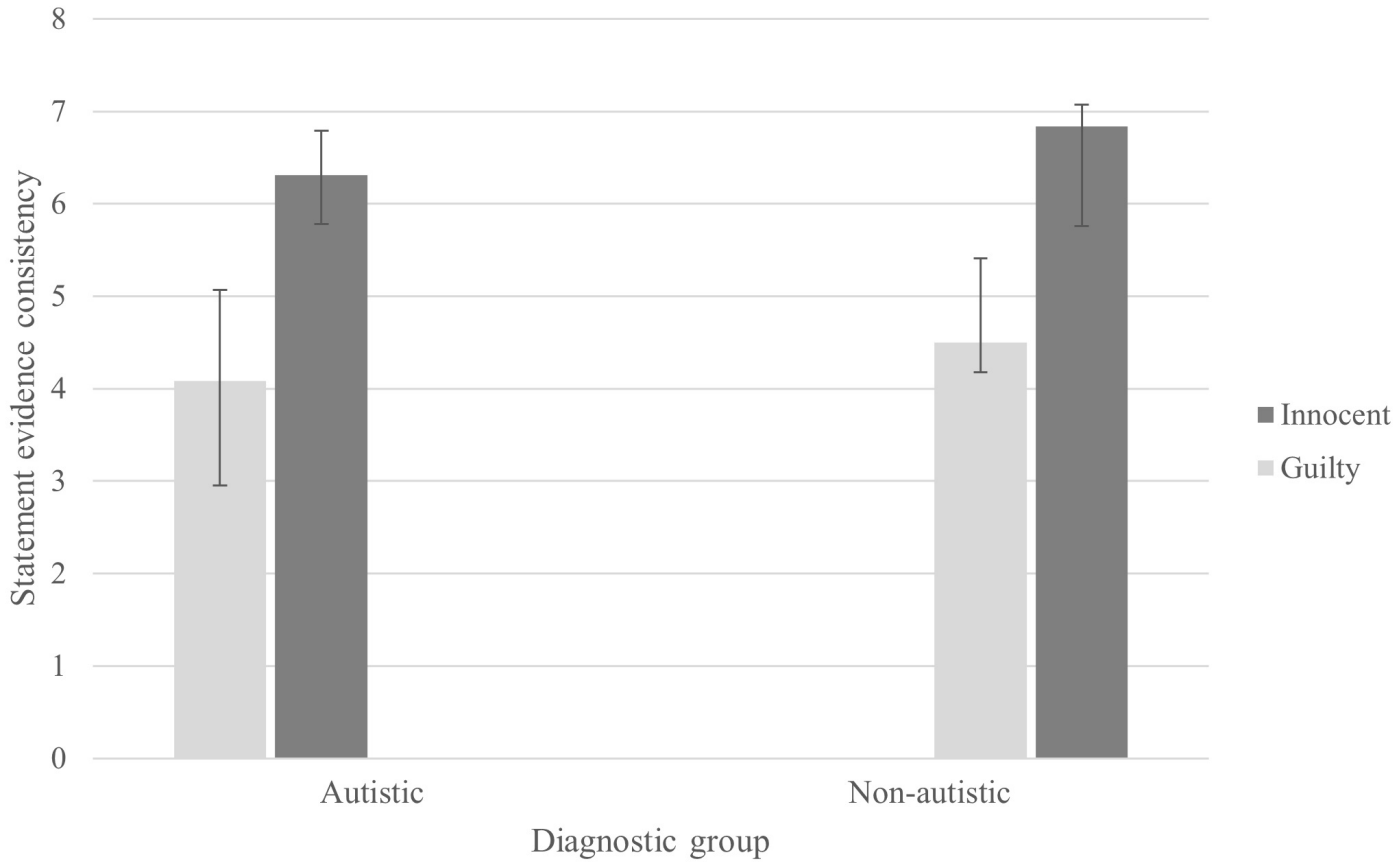
Mean statement evidence consistency scores for autistic and non-autistic groups in innocent and guilty conditions (with BCa 95% confidence error bars).

There was a significant main effect of Condition *F*(1, 59) = 33.65, *p* < 0.001, partial *η*^2^ = 0.363, in which innocent participants’ accounts (*M =* 6.57) were significantly more consistent with the available evidence than guilty participants’ (*M* = 4.29), *M* difference = 2.28, BCa 95% CI (1.69, 3.03). There was no effect of Group or Group X Condition interaction (*ps >* 0.221, *η*^2^s < 0.025). See Figure 2.

#### Extricating information

**Figure 3:**
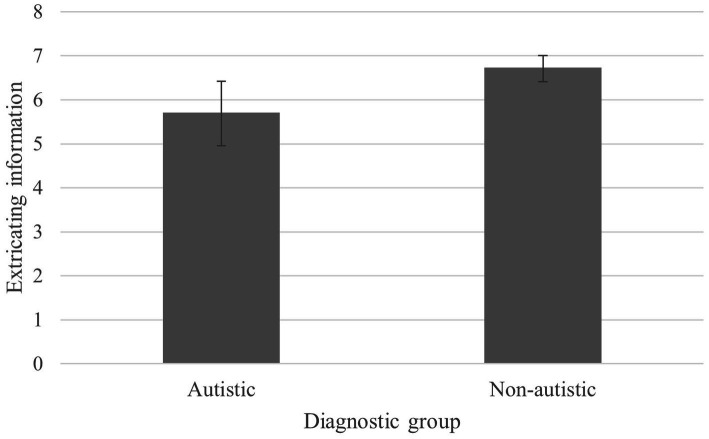
Mean extricating information scores for autistic and non-autistic groups in the innocent condition (with BCa 95% confidence error bars).

Innocent autistic participants drew upon significantly fewer extricating details (*M =* 5.71) than innocent non-autistic participants (*M =* 6.73), *t*(30) = −2.45, *p* = 0.012, *d* = −0.826, *M* difference = −1.03, BCa 95% CI (−1.87, −0.26). See Figure 3.

#### Investigation-relevant information (IRI)

**Figure 4:**
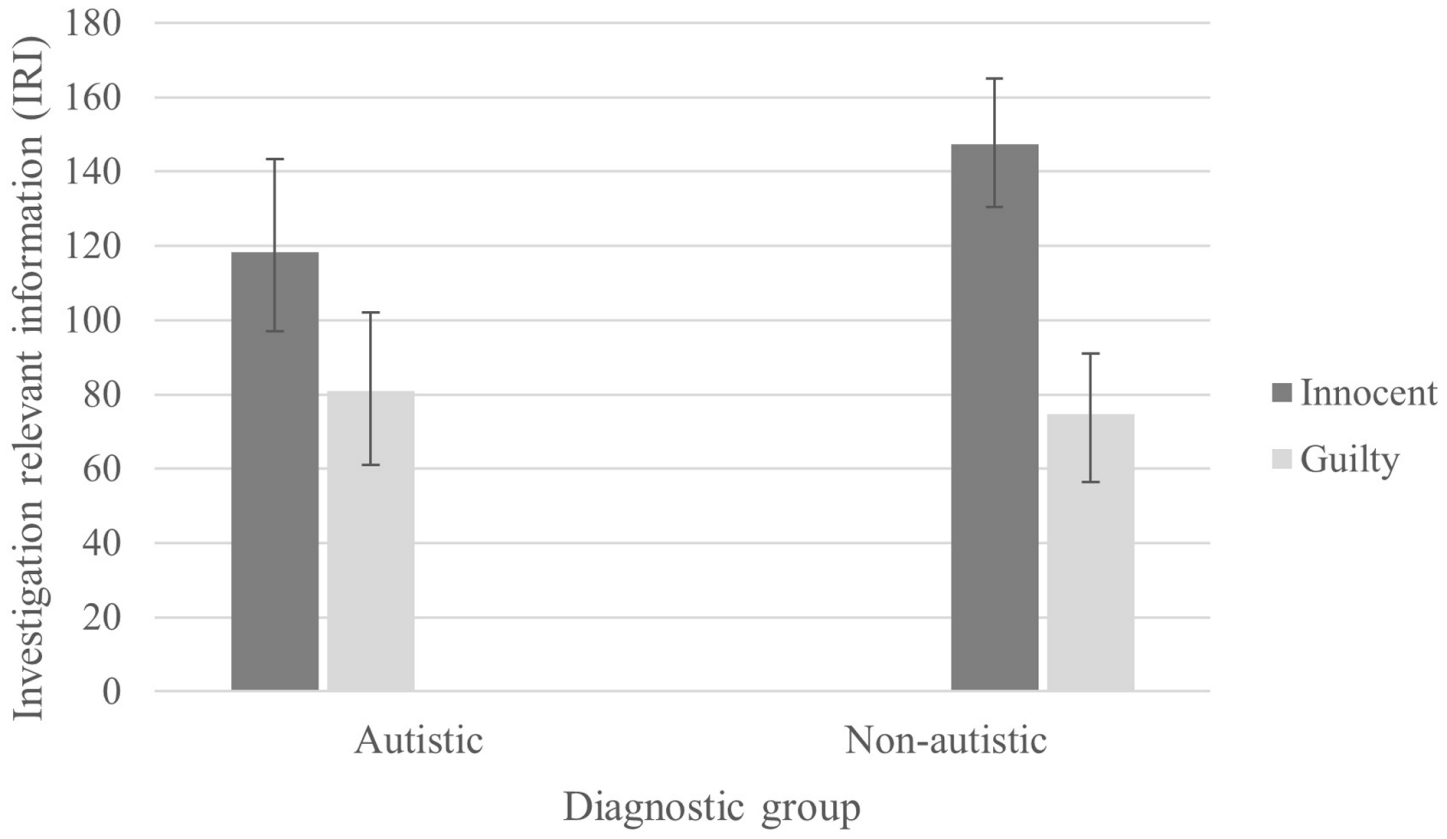
Mean IRI scores for autistic and non-autistic groups in innocent and guilty conditions (with BCa 95% confidence error bars).

There was a significant main effect of Condition *F*(1, 59) = 29.96, *p* < 0.001, partial *η*^2^ = 0.314, for the proportion of IRI provided by participants, whereby innocent participants (*M =* 132.74) provided more detailed accounts than guilty participants (*M =* 77.72), *M* difference = 55.01, BCa 95% CI (30.63, 78.43). There was no significant effect of or Group X Condition interaction (*ps* > 0.079, partial *η*^2^s < 0.051). See Figure 4.

### Interview experience

Finally, we compared autistic and non-autistic participants’ interview experience (see Table 3).

#### Difficulty of questioning

Autistic participants reported that they found the interview questions significantly more difficult (*M =* 4.25) than non-autistic participants (*M =* 3.16), *F*(1, 59) = 6.97, *p* = 0.011, partial *η*^2^ = 0.106, *M* difference = 1.09, BCa 95% CI (0.17, 2.01). There was no effect of Condition or Group X Condition interaction (*ps* > 0.404, *η*^2^s < 0.012).

#### Interview anxiety

Autistic participants found the interview significantly more anxiety inducing (*M* = 4.79) than non-autistic participants (*M =* 3.95), *F*(1, 59) = 4.41, *p* = 0.040, partial η^2^ = 0.069, *M* difference = 0.84, BCa 95% CI (0.07, 1.58). There was no effect of Condition or Group X Condition interaction (*p >* 0.06, η^2^ < 0.069).

#### Perception of support

Autistic participants felt significantly less supported (*M =* 4.44) by the interviewer than non-autistic participants (*M =* 5.30), *F*(1, 59) = 5.62, *p* = 0.021, partial *η*^2^ = 0.087, *M* difference = −0.86, BCa 95% CI (−1.60, −0.14) There was no main effect of Condition or Group X Condition interaction (*ps >* 0.292, *η*^2^s < 0.019).

## Discussion

In the present study, we examined verbal deception cues displayed by autistic adults during a mock-police suspect interview. We found that autistic mock-suspects displayed similar verbal deception cues (in terms of statement-evidence consistency and investigation-relevant information) to the non-autistic group regardless of whether they were telling the truth or being deceptive. To our knowledge this was the first study in which autistic adults (without co-occurring intellectual disability) actively demonstrated the ability to tell verbal lies (see Bagnall et al., 2022). Specifically, the degree to which mock-suspects lied through omitting incriminating actions during the VE task (therefore contradicting the incriminating evidence) was similar regardless of diagnostic group. Further, a similar proportion of investigation-relevant information (IRI) was reported by autistic and non-autistic mock-suspects who lied, with both diagnostic groups providing sparser verbal accounts than mock-suspects who told the truth. This is consistent with reality monitoring theory (Johnson and Raye, 1981), in which information derived from *internal sources* (e.g., imagination) is less detail-rich than information from *external sources* (e.g., experienced events). These findings therefore suggest an at least surface-level consistency between autistic and non-autistic verbal deception.

However, we also found that *innocent* autistic-mock-suspects displayed verbal cues associated with deception. Consistent with findings from Young and Brewer (2020), autistic-mock-suspects who told the truth reported fewer items of verifiable, extricating information to support their innocence. This is concerning given that providing fewer verifiable details during interview is not only consistent with liars’ strategies to avoid disprovable claims (Nahari, 2018), it also makes veracity judgments more difficult (Porter and Salvanelli, 2020) and may narrow the options for further investigation and elimination from enquiries (College of Policing, 2021). As such, our findings emphasize that investigative interviewers should be cautious when interpreting gaps or missing elements in autistic suspects’ accounts. Indeed, previous studies report that expressive language capacity predicts autistic adults’ verbal specificity during interviews (Norris and Maras, 2022) and ToM (ability to take others’ perspectives) relates to their likelihood of providing extricating innocence-supporting detail (Young and Brewer, 2020). Consequently, more supportive interview techniques appear necessary to help autistic suspects report all information relevant to an investigation (more on this point later in the “Discussion”).

Contrary to our expectations, autistic mock-suspects’ statements were not significantly less consistent with the seven pieces of incriminating evidence nor contained significantly less IRI than those of non-autistic mock-suspects. It is of note that innocent autistic-mock-suspects’ statement-evidence consistency was indeed lower than the non-autistic group (with a small effect size of partial *η*^2^ = 0.017), as was proportion of IRI (a medium effect size of partial *η*^2^ = 0.066). The present study was powered to detect medium-large effects of interview performance, meaning that a larger sample may have been necessary to detect these smaller effects. Overall, however, our findings indicate that autistic adults may display certain verbal deception cues when telling the truth during police suspect interviews.

Further, autistic participants (in both innocent and guilty conditions) found interview questions harder to answer, felt more anxious and perceived the interview as less supportive to their needs. While investigative interviews elicit anxiety in neurotypical populations (Vanderhallen et al., 2011) this may be particularly problematic for autistic suspects. Elevated anxiety is associated with poorer executive functioning in autistic adolescents (Hollocks et al., 2014), and broader socio-cognitive processing difficulties (Velikonja et al., 2019) may impact autistic suspects’ ability to provide best evidence. It should be noted that, although autistic participants reported significantly poorer interview experiences than non-autistic participants, average scores still tended to fall in a ‘neutral’ rating. However, the mock-interviewer was specifically trained and instructed to adopt an encouraging and non-confrontational questioning style. Interviews with vulnerable suspects carried out during genuine investigations are often less accommodating. Inappropriate (e.g., forced choice) questions and minimisation tactics have been found to be more commonly used with suspects who have mental health conditions than suspects without such conditions (Farrugia and Gabbert, 2020b). Autistic people have also described feeling overwhelmed by the frequency and length of real-life suspect interviews, as well as difficulty concentrating on questions and experiencing pressure from investigators (Allen et al., 2008). Our present findings therefore likely underplay the difficulty of a real-life suspect interview for an autistic person and the degree to which subsequent verbal behaviors (associated with deception) may be exacerbated. Future research should further investigate the experience of police suspect interviews for autistic people, and the factors which contribute to atypical behavior as well as the elicitation of accurate and reliable information.

It should also be noted that increased anxiety may potentially contribute to autistic people displaying stress-adaptive (though atypical) behaviors during a suspect interview. For example, autistic people (automatically and voluntarily) use gaze aversion and repetitive movement to self-regulate hyperarousal (Collis et al., 2022; Stuart et al., 2022). Concerningly, these nonverbal behaviors are also stereotypically associated with deception (Hartwig and Bond, 2011; Vrij, 2019). Lim et al. (2022) examined whether the presence of specific autism-typical verbal, paraverbal and nonverbal behaviors (gaze aversion, repetitive movement, literal interpretation of figurative language, poor reciprocity and flat affect) predicted truthful autistic mock-suspects as being (incorrectly) perceived as deceptive. Autistic mock-suspects were rated by observers as more deceptive and less credible than non-autistic controls, though none of the hypothesized autism-typical behaviors predicted deception judgments (nor did behaviors significantly differ in prevalence between diagnostic groups). Understanding which verbal, paraverbal and nonverbal characteristics displayed by autistic people are most influential for inaccurate deception judgments, and how these may be exacerbated by police suspect interviews, is a key direction for future research.

The present research is not without limitations. Our participant sample was of above average intelligence (based upon IQ scores), and as such the autistic group is not reflective of the full heterogeneity of the autism spectrum. Indeed, it is estimated that between 13 to 20% of autistic people have co-occurring intellectual disability (Ghirardi et al., 2018; Rydzewska et al., 2019). Given that people with intellectual disabilities may be overrepresented in the CJS (Hellenbach et al., 2017; Chester, 2018), better understanding of specific vulnerabilities for autistic people with co-occurring developmental conditions during investigative interviews is needed. However, given the greater social and cognitive difficulties associated with intellectual disability (Smith et al., 2020), the issues raised in the present study may only be more pronounced for autistic people with co-occurring intellectual disability. It is also important to acknowledge that, unlike in a ‘real’ investigative interview in the UK, autistic mock-suspects were not entitled to support *via* an Appropriate Adult nor legal advisor (Home Office, 1984/2008) either of which may have helped improve interview performance and reduce anxiety. Though as appropriate support is often not provided to autistic adults in custody (Slavny-Cross et al., 2022), our findings emphasise the vulnerabilities of autistic suspects when support is absent.

The potential effects of employing a virtual environment (VE) paradigm should also be considered. It is possible that undertaking a mock-crime or a non-criminal task in a VE (rather than *via* an ‘in-person’ task) contributed to participants perceiving the task and subsequent mock-suspect interview as more simulative. In which case, guilty participants may have perceived their mock-crime to be less transgressive and innocent participants felt it less important they convey their innocence. However, previous studies have shown computer game-based (mock-crime and non-criminal) tasks to be effective when participants are required to generate truthful and deceptive accounts during a subsequent mock-investigative interview (e.g., Dando and Bull, 2011). Further, virtual recreations of tasks such as public speaking can induce comparable levels of stress response to real-life equivalent tasks (Kothgassner et al., 2016) and such immersive technologies often provide valid alternatives to *in vivo* (real-life) exposure (Wechsler et al., 2019; Liberatore and Wagner, 2021). It should be noted that we used a desktop-based VE paradigm in the present study, and VEs presented on head-mounted displays (e.g. 3D virtual reality) can produce greater spatial presence and immersion (Shu et al., 2019). The potential effects of using a desktop-based VE (rather than 3D virtual reality) in the present study is unclear. A more realistic criminal and non-criminal task (i.e., in 3D virtual reality) may elicit a greater sense of ecological validity and influence mock-suspects’ perception of a subsequent mock-suspect interview (e.g., feel a greater desire to convey their innocence). However, given that autistic participants in the present study experienced the mock-interviews as more demanding than non-autistic participants, such group differences may only be more pronounced if a more realistic simulation were used. Future research may therefore benefit from presenting VEs using head-mounted displays (i.e., in 3D virtual reality) to further examine potential vulnerabilities for autistic people during suspect interview settings.

These limitations notwithstanding, the present research has several implications for practice. Our findings highlight the additional complexity for investigators when interviewing autistic suspects, as verbal deception cues may be displayed whether the interviewee is truthful or lying. Existing witness and suspect interview models provide future directions for more supportive practice to benefit both interviewer and interviewee. The Witness-Aimed First Account (WAFA) approach reduces social and cognitive demand through autistic mock-witnesses generating segmented event memories prior to ‘free recall’, resulting in more detailed and accurate accounts while also being making autistic mock-witnesses feel more socially comfortable (Maras et al., 2020). In turn, this approach may aid recall of relevant, verifying information while reducing stress-induced paraverbal and nonverbal behaviors (i.e., deception cues). Further, the ‘Model Statement’—an example of a detailed statement on an unrelated topic presented to a suspect pre-interview (Leal et al., 2015)—may help account for autistic peoples’ difficulty in gauging relevance and quantity of required information. An interviewer being more explicit and specific about what is expected of autistic suspects during interview may assist the suspect’s understanding of questioning. Further research is needed to ascertain the validity of such models for detecting truth and lies in autistic suspects.

However, adaptations to interview practice with autistic suspects are dependent upon pre-interview identification of ‘vulnerability’. Although custody staff and interviewing officers are guided to consider signs of potential vulnerability (College of Policing, 2022), an autistic suspect may not be correctly identified due to a lack of specific questions about autism during the ‘booking in’ phase in custody (Sims, 2017) or because a detainee chooses not to disclose being autistic out of concern of stigma (Crane et al., 2016). A lack of awareness of a suspect’s autism may lead to harsher interpretations of their behavior during interview (Maras et al., 2019; Logos et al., 2021; Lim et al., 2022). Custody staff should therefore make additional efforts to identify potential autistic detainees (Holloway et al., 2022) and interviewers should be conscious of avoiding a guilt-presumptive questioning style (Lidén et al., 2018). Despite PACE guidelines (section 11C) in the UK stating that vulnerable suspects’ accounts may be inadvertently “unreliable” or “misleading,” and that “corroboration of any facts admitted” should be obtained and an appropriate adult provided, there is little further specific guidance for interviewers relating to this issue. Extending this guidance with evidence-based examples highlighting the heterogeneity of autistic verbal, paraverbal and nonverbal behavior and embedding it in policy and training is an important future direction for CJS practice.

## Conclusion

In the present study, we found that investigative interviews are more socially and cognitively demanding for autistic than neurotypical mock-suspects. In addition, verbal cues associated with deception can be displayed by autistic mock-suspects even when truthful. The development of autism-focused suspect interview techniques is therefore crucial to resolve the (interrelated) issues of interviewee welfare and provision of best evidence. Discriminating between *difficulty* and *deception* in autistic suspects’ interview accounts is a challenging though necessary task for researchers and practitioners alike.

## Data availability statement

The raw data supporting the conclusions of this article will be made available by the authors, without undue reservation.

## Ethics statement

The studies involving human participants were reviewed and approved by Psychological Research Ethics Committee at the University of Bath. The participants provided their written informed consent to participate in this study.

## Author contributions

RB, AR, MB, MO, and KM contributed to the conceptualization and design of the study. RB and AC collected and coded the data. RB performed the statistical analysis and wrote the first draft of the manuscript. All authors contributed to the article and approved the submitted version.

## Funding

This work was conducted as part of PhD research undertaken by RB, funded by the Economic and Social Research Council (2096910).

## Conflict of interest

The authors declare that the research was conducted in the absence of any commercial or financial relationships that could be construed as a potential conflict of interest.

## Publisher’s note

All claims expressed in this article are solely those of the authors and do not necessarily represent those of their affiliated organizations, or those of the publisher, the editors and the reviewers. Any product that may be evaluated in this article, or claim that may be made by its manufacturer, is not guaranteed or endorsed by the publisher.

## Footnotes

^1^The pattern of results remained the same when statistical analyses were performed with and without these participants in the dataset.

^2^Gaming experience was assessed by participants rating how often they play computer games on a 1–5 Likert scale: 1 = Never; 2 = Once or twice per month; 3 = Less than 50% of days; 4 = 50% or more of days; 5 = Every day.

^3^Participants first received a brief training session for how to navigate the VE (e.g., mouse/keypad buttons) in a separate virtual location to the main *criminal* or *non-criminal* task.

^4^The UK uses the PEACE model interview, P, Planning and preparation; E, Engage and explain; A, Account, clarify and challenge; C, Closure; E, Evaluation (College of Policing, 2022). In the present study, only the Account, clarify and challenge phase was implemented.
