# Supplementary material for: Police suspect interviews with autistic adults: The impact of truth telling versus deception on testimony
Source: Front Psychol. 2023 Mar 22;14:1117415. doi: 10.3389/fpsyg.2023.1117415 (PMC10074602; doi:10.3389/fpsyg.2023.1117415)
Supplement: Supplementary file 1 [file Data_Sheet_1.PDF]

Virtual city environment – Innocent Condition	Virtual city environment – Guilty Condition
**Pre-screen:** *“You have just left home to take your dog for a walk. Your housemate James will be sending you text messages to remind you to do some tasks. You are also planning on meeting a friend called Gary later, and they will text you to arrange this. Please follow all text message instructions when they appear on screen.*<br>*When you are ready to start, please press the spacebar.”*	**Pre-screen:** *“You have just left home to take your dog for a walk. Your housemate Stan will be sending you text messages to remind you to do some tasks. You have planned to burgle a house with an accomplice called Fred, and they will text you to arrange this. Please follow all text message instructions when they appear on screen.*<br>*When you are ready to start, please press the spacebar.”*
**Text from James (housemate):** *“Take the dog for a walk around the park and come back to number 5.”*	**Text from Stan (housemate):** *“Take the dog for a walk around the park and come back to number 5.”*
**ACTION 1:** User walks dog around park then takes dog home *(passes witness at edge of park)*	**ACTION 1:** User walks dog around park then takes dog home *(passes witness at edge of park)*
**Text from James (housemate):** *“Don’t forget that letter you promised to post – please post in the letterbox at the end of the street”*	**Text from Stan (housemate):** *“Don’t forget that letter you promised to post – please post in the letterbox at the end of the street”*
**ACTION 2:** User walks to letterbox, posts letter *(passes witness outside shop)*	**ACTION 2:** User walks to letterbox, posts letter *(passes witness outside shop)*
**Text from Gary (friend):** *“Hey, take the bus to come meet me near my house and I’ll pick you up”*	**Text from Fred (accomplice):** *“OK, take the bus to the location and we’ll send instructions”*
**ACTION 3:** Bus arrives, User gets on bus.<br>*“You are now travelling to meet your friend”*	**ACTION 3:** Bus arrives, User gets on bus.<br>*“You are now travelling to the burglary location”*
**Virtual suburban environment – Innocent Condition**	**Virtual suburban environment – Guilty Condition**
**ACTION 4:** User gets off bus at bus stop *(CCTV at bus stop)*	**ACTION 4:** User gets off bus at bus stop *(CCTV at bus stop)*
**Text from Gary (friend):** *“Hey, I’m running a few minutes late, take a walk around the pond in the park – it’s nicer there”*	**Text from Fred (accomplice):** *“Make sure you are well hidden, wait by the pond in the park”*
**ACTION 5:** User walks to pond in park *(drops glove in park)*	**ACTION 5:** User walks to pond in park *(drops glove in park)*
**Text from Gary (friend):** *“Sorry, traffic is terrible, why don’t you take a walk around the block while you wait for me. Go past the red letter box on the corner of the park and then turn left by the boxes”*	**Text from Fred (accomplice):** *“Take a walk around the block to check for potential witnesses. Go past the red letter box on the corner of the park and then turn left by the boxes”*
**ACTION 6:** User walks around side street at back of houses, then up side-street *(passes witness in bedroom window)*	**ACTION 6:** User walks around side street at back of houses, then up side-street *(passes witness in bedroom window)*
**Text from Gary (friend):** *"Sorry, the traffic is terrible. Can you check if my laptop is still on the backseat of the white car (outside house number 1)?”*	**Text from Fred (accomplice):** *“OK, someone’s just left, check that the white car outside house number 1 is empty and we’ll send the next instructions.”*

**ACTION 7:** User walks to car outside house *(witness is further down the road)*	**ACTION 7:** User walks to car outside house *(witness is further down the road)*
**Text from Gary (friend):** *"Oh, it's not there? OK, could you run into House number 1 and check if it's there? You can go in through the back garden gate. I can't remember where I left it, so please check everywhere including upstairs …."*	**Text from Fred (accomplice):** *"Enter House number 1 through garden gate and find the laptop. We don't know where this will be, check everywhere including upstairs. Find it and steal it."*
**ACTION 8:** User enters house through garden gate, explores house, finds laptop upstairs but doesn't take it *(leaves shoe print on stairs)*	**ACTION 8:** User enters house through garden gate, explores house, finds laptop upstairs and picks it up *(leaves shoe print on stairs)*
**Text from Gary (friend):** *"Ah it's on the bed? Great. OK, I also forgot my key, can you grab it for me? It's by the front door. Then head to the park and I'll pick you up."*	**Text from Fred (accomplice):** *"OK, take the key hanging by the front door and head to the park – someone will pick you up there."*
**ACTION 9:** User goes downstairs *(footprint left on stairs is visible),* takes key, exits house and walks to park.	**ACTION 9:** User goes downstairs *(footprint left on stairs is visible),* takes key, exits house and walks to park.
**Text from Gary (friend):** *"Sorry, I'm going to be really late, please just take the bus home and take the stuff with you, I'll pick it up later."*	**Text from Fred (accomplice):** *"OK, change of plan – take the bus home and take the stuff with you, someone will come and pick it up later."*
**ACTION 10**: User goes to bus *(CCTV at bus stop),* takes bus back to city. *"You are now travelling back home"*	User goes to bus **(CCTV at bus stop)**, takes bus back to city. *"You are now travelling back home"*
**Virtual city environment – Innocent Condition**	**Virtual city environment – Guilty Condition**
**ACTION 11:** User gets off bus and walks home.	**ACTION 11:** User gets off bus and walks home.
Virtual Environment fades to black. *"You have now gone home and you stay there for the rest of the day and evening. Please press the spacebar to exit the virtual environment."*	Virtual Environment fades to black. *"You have now gone home and you stay there for the rest of the day and evening. Please press the spacebar to exit the virtual environment."*
