# Supplementary material for: Police suspect interviews with autistic adults: The impact of truth telling versus deception on testimony
Source: Front Psychol. 2023 Mar 22;14:1117415. doi: 10.3389/fpsyg.2023.1117415 (PMC10074602; doi:10.3389/fpsyg.2023.1117415)
Supplement: Supplementary file 2 [file Data_Sheet_2.PDF]

Interviewer script

Available evidence (numbers in red relate to evidence in relation to burglary):

	Known information	Source
	**In home area:**	
	Suspect took dog for walk along edge of park	Eyewitness
	Suspect posted letter in post-box	Eyewitness
	**In burglary area:**	
**1**	Suspect travelled from home area by bus and got off the bus on the street of the burgled property	CCTV at bus stop
**2**	Suspect remained in park near burgled property for short while	Glove found in park matching suspects’
**3**	Suspect walked the side streets adjacent to burgled property	Eyewitness
**4**	Suspect looked inside the car parked outside the burgled property	Eyewitness
**5**	Suspect entered the property at which the burglary took place	Footprint on stairs matching suspects’
**6**	Suspect got back on bus in burglary area and left	CCTV at bus stop
	**In home area:**	
**7**	Key to burgled property found in suspects’ home	Police search of property

Interview structure:

- Introduction
- Caution the suspect
- Grounds for arrest
- Obtain initial account
  - Make notes of initial account
  - Segment into different topics (locations/actions)
  - Update evidence checklist
- Probe questioning based on topics
  - Update evidence checklist
- Questions about incriminating evidence
  - Adapt questions based on evidence checklist

- Interview close

Introduction:

"Interview with suspect number (use participant number)"

"This interview is being audibly/visibly recorded using a secure digital network."

"I am (state name)"

"Please confirm that there are no other persons present" (Suspect to reply)

"The date is (state day, date, month of test)"

"The time by the Interview clock is … (state time)"

"This interview is being conducted in Interview Room 1.26"

Caution the suspect:

"You do not have to say anything. But it may harm your defence if you do not mention when questioned something which you later rely on in court. Anything you do say may be given in evidence."

Grounds for arrest:

"The grounds for your arrest are that on the (x date) at (x time)" a property in the (x) area was illegally entered with the intent of theft and a number of items were stolen from the property, including a laptop. You've been arrested on suspicion of theft."

Expectations:

"During interview I will expect you to listen carefully to my questions.

I will be giving you time to think.

I will be expecting you to give me as much detail as possible ... please don't leave anything out.

Do you have any questions for me at this stage?"

Obtain initial account:

"Take me through what you were doing between (X time) on (X date). Please describe this to me in as much detail as you can."

Interviewer records brief notes on suspects' main points during initial account. Aim to write but maintain eye-contact, nodding, encouraging. While writing put these into separate locations/actions:

Initial account record:

**Suspects initial account**	**Segment into discrete locations/actions for probe questioning**	
	1	
	2	
	3	
	4	
	5	
	6	
	7	
	8	

Update evidence checklist:

When the suspect has finished giving their initial account, add ticks or crosses indicating whether the known information was included in their initial account.

	**Known information**	**Source**	**Mentioned in initial account?**	**Mentioned in probe questions?**

	**In home area:**			
	Suspect took dog for walk along edge of park	Eyewitness		
	Suspect posted letter in post-box	Eyewitness		
	**In burglary area:**			
**1**	Suspect travelled from home area by bus and got off the bus on the street of the burgled property	CCTV at bus stop		
**2**	Suspect remained in park for short while	Glove found in park matching suspects'		
**3**	Suspect walked the side streets adjacent to burgled property	Eyewitness		
**4**	Suspect looked inside the car parked outside the burgled property	Eyewitness		
**5**	Suspect entered the property at which the burglary took place	Footprint on stairs matching suspects'		
**6**	Suspect got back on bus in burglary area and left	CCTV at bus stop		
	**In home area:**			
**7**	Key to property found in suspects' home	Police search		

Probe questions based on topics:

When suspect responds to the probe questions (below), also ask one or two brief follow up clarification questions based on their answers (not referencing any incriminating info unless they mention it).

(example) "OK, so you say you took the **dog for a walk**, tell me more about that."

- *Example clarification question (if they mention seeing someone whilst on their walk): "What did this person look like?"*
- *Example clarification question (if they mention taking the dog back home): "What time did you take the dog back home?"*

Update evidence checklist:

During participants' answers to probe questions, add ticks or crosses indicating whether the known information is included in their answers.

Evidence disclosure:

Before asking these questions, check whether there is known information <u>not</u> mentioned by the suspect at initial account <u>and</u> probe questions stages. If mentioned by the suspect at either stage, use the 'mentioned / explained' question. If not mentioned by suspect at this point, use the 'not mentioned / not explained' question:

"Thank you for answering my questions so far. I am now going to talk you through the evidence that currently implicates you in the burglary."

Evidence 1 mentioned / explained:

- "As you described, you were at the bus stop near the property in question at 2.00pm. Now, this is incriminating as it tells us you were in the area on the day and approximate time the burglary was committed. Do you have anything further you wish to add?"

Evidence 1 not mentioned / not explained:

- "Now, I'm a bit confused as there are some parts of your account which don't match the evidence we hold. You were witnessed at the bus stop near the property at 2.00pm, which is incriminating as it tells us you were in the area on the day and approximate time the burglary was committed. How would you explain this?"

Evidence 2 mentioned / explained:

- "As you described, you were in the park near the property in question at 2.00pm. This is further incriminating evidence as it tells us you were in the area on the day and approximate time the burglary was committed. Do you have anything further you wish to add?"

Evidence 2 not mentioned / not explained:

- "There are some (more) parts of your story which don't appear to match up with the evidence we hold. A glove belonging to you was found in the park near the burgled property. This is incriminating as it tells us you were in the area on the day and approximate time the burglary was committed. How would you explain this?"

Evidence 3 mentioned / explained:

- "As you've described, you took a walk around the streets adjacent to the property in question at approximately 2pm. This is further incriminating evidence as it tells us you

were in the area on the day and approximate time the burglary was committed. Do you have anything further you wish to add?"

Evidence 3 not mentioned / not explained:

- "There are some (other) parts of your story which don't appear to match up with the evidence we hold. Someone matching your description was witnessed in the streets adjacent to the property. This is incriminating as it tells us you were in the immediate vicinity of the property on the day the burglary was committed. What is your explanation for this?"

Evidence 4 mentioned / explained:

- "As you've described, you were outside the property in question at approximately 2pm. This is further incriminating evidence as it tells us you were in the area on the day and approximate time the burglary was committed. Do you have anything further you wish to add?"

Evidence 4 not mentioned / not explained:

- "There are some (other) parts of your story which don't appear to match up with the evidence we hold. Someone matching your description was witnessed outside the property, looking into the car parked outside. This is incriminating as it tells us you were outside the property on the day the burglary was committed. What is your explanation for this?"

Evidence 5 mentioned / explained:

- "As you've described, you were inside the property in question at approximately 2pm. This is further incriminating evidence as it tells us you were in the property on the day and approximate time the burglary was committed. Do you have anything further you wish to add?"

Evidence 5 not mentioned /not explained:

- "Now, I'm a bit confused as there are some parts of your account which don't match the evidence we hold. A footprint matching yours was found on the stairs to the third floor. This is incriminating as it tells us you were inside the property on the day the burglary was committed. What is your explanation for this?"

Evidence 6 mentioned / explained:

- "As you've described, you got back on the bus near the property. This is further incriminating evidence as it tells us you left the area shortly after when we believe the burglary took place. Do you have anything further you wish to add?"

Evidence 6 not mentioned / not explained:

- "Now, I'm a bit confused as there are some (other) parts of your account which don't match the evidence we hold. Someone matching your description was witnessed getting back on the bus near the property. This is incriminating as it tells us you left the area shortly after when we believe the burglary took place. What is your explanation for this?"

Evidence 7 mentioned / explained:

- "As you've described, you took the key and this was found in your property. This is incriminating as it tells us you took an item from the property that was burgled that day. Do you have anything further you wish to add?"

Evidence 7 not mentioned / not explained:

- "There are some (other) parts of your story which don't appear to match up with the evidence we hold. The key to the property in question was found at your home address. This is incriminating as it tells us you took an item from the property that was burgled that day. What is your explanation for this?"

Final questions:

- "Did you enter the property and steal the key?"

- "Did you enter the property and steal the laptop?"

Interview closure:

"Is there anything you wish to further add to your statement at this time?"
"Interview terminated at (time)".

Interview ends. Thank participant.
